# Supplementary material for: Heterologous Prime-Boost Vaccination with Commercial FMD Vaccines Elicits a Broader Immune Response than Homologous Prime-Boost Vaccination in Pigs
Source: Vaccines (Basel). 2023 Feb 25;11(3):551. doi: 10.3390/vaccines11030551 (PMC10058820; doi:10.3390/vaccines11030551)
Supplement: Supplementary file 1 [file vaccines-11-00551-s001.zip › vaccines-2192269-supplementary.pdf]

Table S1. The ELISA results of individual pigs assayed by VDPro FMDV type O Ab b-ELISA kit before FMDV vaccination (7 weeks of age).

A-A		A-B		A-C		B-B		B-A		B-C		C-C		C-A		C-B	
No.	PI (%)[1]	No.	PI (%)	No.	PI (%)	No.	PI (%)	No.	PI (%)	No.	PI (%)	No.	PI (%)	No.	PI (%)	No.	PI (%)
501	94.6	101	94.8	112	94.8	801	92.9	201	97.5	212	72.0	901	77.9	301	32.3	401	27.5
502	96.8	102	96.7	113	91.8	802	82.6	202	96.9	213	60.7	902	92.9	302	97.5	402	96.2
503	77.4	103	95.2	114	89.6	803	78.4	203	99.6	214	89.9	903	79.2	303	35.2	403	86.1
504	88.8	104	76.5	115	94.5	804	91.8	204	71.7	215	96.2	904	70.7	304	88.7	404	91.3
505	80.8	105	94.3	116	95.5	805	46.9	205	84.8	216	93.1	905	90.6	305	58.8	405	49.0
506	77.5	106	95.4	117	95.1	806	90.3	206	93.4	217	85.1	906	80.8	306	99.2	406	83.0
507	72.6	107	95.1	118	90.7	807	11.6	207	95.6	218	78.0	907	85.0	307	34.4	407	93.8
508	80.8	108	93.6	119	96.5	808	92.1	208	88.7	219	87.3	908	90.4	308	77.5	408	89.9
509	70.6	109	92.4	120	96.5	809	82.5	209	96.1	220	72.4	909	88.9	309	84.9	409	53.0
510	12.8	110	94.0	121	96.2	810	89.6	210	77.3	221	96.2	910	87.5	310	77.7	410	81.8
511	81.9	111	91.7	122	94.5	811	91.7	211	97.3	222	94.8	911	10.9	311	80.8	411	97.1
512	77.4	601	78.2	612	35.8	812	67.7	701	53.3	712	83.5	912	81.4	312	75.7	412	79.3
513	90.3	602	88.8	613	81.1	813	94.9	702	25.2	713	98.0	913	77.5	313	83.8	413	88.7
514	89.1	603	94.9	614	83.9	814	49.7	703	86.4	714	81.2	914	82.9	314	91.0	414	83.9
515	96.6	604	91.7	615	75.5	815	75.6	704	48.2	715	96.0	915	65.2	315	75.1	415	32.8
516	84.3	605	2.1	616	82.3	816	91.1	705	86.9	716	52.1	916	82.7	316	97.8	416	84.0
517	20.2	606	90.5	617	17.4	817	86.1	706	84.4	717	35.8	917	86.0	317	33.1	417	75.9
518	71.3	607	17.8	618	92.9	818	85.3	707	86.0	718	84.4	918	91.3	318	76.5	418	33.8
519	76.8	608	76.2	619	85.5	819	87.2	708	80.2	719	46.9	919	1.6	319	78.6	419	91.2
520	92.9	609	86.8	620	87.8	820	91.1	709	78.6	720	77.3	920	83.5	320	36.7	420	40.2
521	88.1	610	91.6	621	94.0	821	67.3	710	56.2	721	85.7	921	64.9	321	50.8	421	88.5
522	70.3	611	86.6	622	47.2	822	13.4	711	83.3	722	43.2	922	81.1	322	76.0	422	84.4
Mean	76.9	Mean	83.0	Mean	82.7	Mean	75.4	Mean	80.3	Mean	77.7	Mean	75.1	Mean	70.1	Mean	74.1
SD[2]	21.3	SD	24.5	SD	21.3	SD	24.3	SD	19.0	SD	18.7	SD	23.6	SD	22.8	SD	22.9

[1] Percent inhibition (%)

[2] Standard deviation
